# Marine Fungal Cerebroside Flavuside B Protects HaCaT Keratinocytes against *Staphylococcus aureus* Induced Damage

**DOI:** 10.3390/md19100553

**Published:** 2021-09-29

**Authors:** Ekaterina A. Chingizova, Ekaterina S. Menchinskaya, Artur R. Chingizov, Evgeny A. Pislyagin, Elena V. Girich, Anton N. Yurchenko, Irina V. Guzhova, Valery V. Mikhailov, Dmitry L. Aminin, Ekaterina A. Yurchenko

**Affiliations:** 1 Laboratory of Bioassays and Mechanism of Action of Biologically Active Substances, G.B. Elyakov Pacific Institute of Bioorganic Chemistry, Russian Academy of Sciences, Prosp. 100 Let Vladivostoku 159, Vladivostok 690022, Russia; chingizova_ea@piboc.dvo.ru (E.A.C.); menchinskaya_es@piboc.dvo.ru (E.S.M.); pislyagin_ea@piboc.dvo.ru (E.A.P.); daminin@piboc.dvo.ru (D.L.A.); 2 Laboratory of Microbiology, G.B. Elyakov Pacific Institute of Bioorganic Chemistry, Russian Academy of Sciences, Prosp. 100 Let Vladi-vostoku 159, Vladivostok 690022, Russia; chingizov_ar@piboc.dvo.ru (A.R.C.); mikhailov@piboc.dvo.ru (V.V.M.); 3 Laboratory of Chemistry of Microbial Metabolites, G.B. Elyakov Pacific Institute of Bioorganic Chemistry, Russian Academy of Sciences, Prosp. 100 Let Vladivostoku 159, Vladivostok 690022, Russia; ev.girich@piboc.dvo.ru (E.V.G.); yurchenkoan@piboc.dvo.ru (A.N.Y.); 4 Institute of Cytology, Russian Academy of Sciences, Tikhoretsky Ave., 4, St. Petersburg 194064, Russia; irina.guzhova@incras.ru; 5 Department of Biomedical Science and Environmental Biology, Kaohsiung Medical University, No.100, Shin-Chuan 1st Road, Sanmin Dist., Kaohsiung City 80708, Taiwan

**Keywords:** *Staphylococcus aureus*, antibacterial activity, anti-inflammatory activity, cerebroside, marine fungi, secondary metabolites, apoptosis, sortase A, biofilm formation

## Abstract

Cerebrosides are glycosylated sphingolipids, and in mammals they contribute to the pro-/anti-inflammatory properties and innate antimicrobial activity of the skin and mucosal surfaces. *Staphylococcus aureus* infection can develop, not only from minor scratches of the skin, but this pathogen can also actively promote epithelial breach. The effect of cerebroside flavuside B from marine sediment-derived fungus *Penicillium islandicum* (Aniva Bay, the Sea of Okhotsk) on viability, apoptosis, total caspase activity, and cell cycle in human epidermal keratinocytes HaCaT line co-cultivated with *S. aureus*, as well as influence of flavuside B on LPS-treated HaCaT cells were studied. Influence of flavuside B on bacterial growth and biofilm formation of *S. aureus* and its effect on the enzymatic activity of sortase A was also investigated. It was found *S. aureus* co-cultivated with keratinocytes induces caspase-depended apoptosis and cell death, arrest cell cycle in the G0/G1 phase, and increases in cellular immune inflammation. Cerebroside flavuside B has demonstrated its antimicrobial and anti-inflammatory properties, substantially eliminating all the negative consequences caused by co-cultivation of keratinocytes with *S. aureus* or bacterial LPS. The dual action of flavuside B may be highly effective in the treatment of bacterial skin lesions and will be studied in the future in in vivo experiments.

## 1. Introduction

*Staphylococcus aureus* (SA) toxicity due to a variety of extracellular toxins; these include the shock syndrome toxin 1, exfoliative toxins, staphylococcal enterotoxins, hemolysins, and leukocidins [1]. SA infection can develop not only from minor scratches of the skin, but this pathogen can also actively contribute to damage to the epithelial breach by α-toxin [2]. Alpha-toxin induces a series of inflammatory events in the target cells and induces inflammasomes, generally at lytic concentrations and probably as part of the events leading to cell death by apoptosis and/or pyroptosis, but some are also observed at sublytic concentrations. Moreover, cytolytic peptides called phenol-soluble modulins have a strong pro-inflammatory and chemotactic effects on neutrophils and keratinocytes at sublytic concentrations. Worldwide incidence of bacterial skin diseases in 2019 was reported by Global Burden Project as 14,684.3 cases per 100,000 of population [3]. It was rarely fatal (0.9 cases per 100,000), but the smallest infection can lead to sepsis if it is unfavorable. In 2017, an estimated 48.9 million incident cases of sepsis were recorded worldwide and 11.0 million sepsis-related deaths were reported, representing 19.7% of all global deaths [4] and SA is one of the main cause of this.

One of the key SA virulence enzymes is a membrane-associated enzyme sortase A (EC 3.4.22.70), which is responsible for the covalent anchoring of many virulent factors of Gram-positive bacteria onto the cell wall. It is well-known that sortase A plays a pivotal role in the pathogenic processes of bacterial SA infection [5]. The lack of sortase A is due to the inability to bind surface proteins that are anchored at their C-terminal ends, and thus surface adhesion is abolished and the process of establishing infection is reduced [6].

Cerebrosides are glycosylated sphingolipids and are crucially important constituents of eukaryotic cells. In mammals, sphingolipids promote pro-/anti-inflammatory properties and innate antimicrobial activity of the skin and mucosal surfaces [7]. Obviously, they perform similar functions in the cells of other eukaryotic organisms, including fungi. It has been reported that more than 50 cerebrosides with antibacterial, anti-inflammatory and others biological activities has been isolated from various fungi [8] and marine-derived microfilamentous fungi are no exception. Chrysogeside B from the halotolerant fungus *Penicillium chrysogenum* PXP-55 showed antimicrobial activity against *Enterobacter aerogenes* [9]. Cerebrosides from the marine tunicate-associated fungus *Penicillium* sp. penicilloside A revealed antifungal activity towards *Candida albicans*, and penicilloside B was active against SA and *Escherichia coli* [10]. Cerebrosides flavuside A and B were isolated from the fungus *Aspergillus flavus* associated with green algae and exhibited mild antibacterial activity against SA, methicillin-resistant SA, and multidrug-resistant SA [11], but this data cannot be used due to absence of an adequate method description.

**Figure 1:**
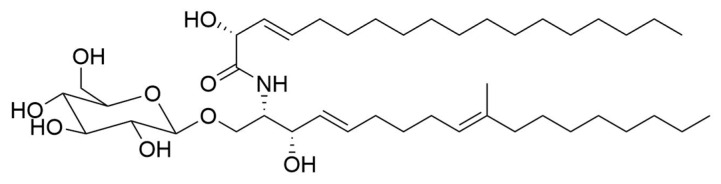
Chemical structure of flavuside B.

Recently we reported the isolation from marine sediment-derived fungus *Penicillium islandicum* and identification of cerebroside flavuside B (Figure 1) having chemical structure (R,E)-2-hydroxy-N-((2S,3S,4E,9E)-3-hydroxy-10-methyl-1-(((2R,3R,4S,5S,6R)-3,4,5-trihydroxy-6-(hydroxymethyl)tetrahydro-2H-pyran-2-yl)oxy)octadeca-4,9-dien-2-yl)octadec-3-enamide [12]. 

The first aim of present work is to study various aspects of anti-staphylococcal properties of flavuside B, such as the influence on the SA bacterial growth, the biofilm formation, and the activity of sortase A enzyme. The direct antibacterial action of the substances may not be enough for the substance to be effective against bacterial infectious cell damage because cytotoxicity of substances and other restrictions may be relevant. In this regard, co-cultivation of human epidermal keratinocytes with various bacteria is used as a model of skin bacterial infections in vitro [13]. Thus, the second aim of this work is to assess the ability of flavuside B to protect human keratinocytes HaCaT from damage induced its co-cultivation with SA. Moreover, the effect of flavuside B on lipopolysaccharide-treated HaCaT cells was studied for verification of its anti-inflammatory properties.

## 2. Results

### 2.1. Antimicrobial Activity of Flavuside B against SA

To understand whether flavuside B has a direct anti-staphylococcal effects, its influence on bacterial growth and biofilm formation in a SA suspension were investigated. The effect of flavuside B up to 100 µM on bacterial growth and biofilm formation of SA is presented in Table 1. 

Flavuside B decreased SA culture growth with an EC_50_ of 99.23 ± 1.12 µM. Inhibition of bacterial growth by 27–28% was detected at a concentration of 25 µM, and it was similar when concentration of flavuside B was reduced 2.5 time. Moreover, flavuside B reduced formation of SA biofilm by 28% at a concentration of 10 µM which was also effective against SA growth. The increase in flavuside B concentration did not result in a greater inhibition of SA biofilm formation (Table 1).

### 2.2. Sortase A Activity Inhibition

The effects of flavuside B on the enzymatic activity of SA sortase A were estimated to make assumptions about a possible mechanism of antibacterial action.

**Figure 2:**
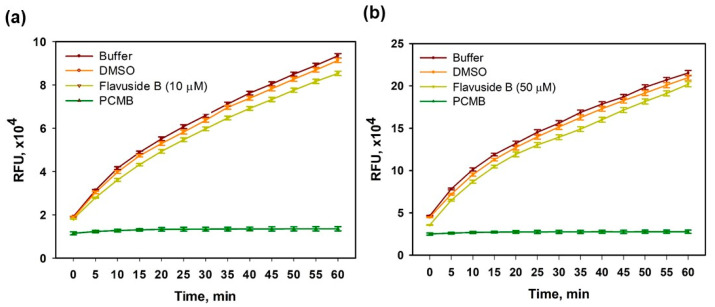
Reaction kinetics of SA sortase A in the presence of flavuside B and selective inhibitor 4-(hydroxymercuri)benzoic acid (PCMB) at the same level of substrate concentration. DMSO (0.1%) did not show significantly inhibitory activity in comparison with PBS. (**a**) Flavuside B was used at a concentration of 10 μM. (**b**) Flavuside B was used at a concentration of 50 μM. All experiments were performed in three independent replicates. The data presented as a mean ± SEM.

Flavuside B significantly inhibited SA sortase A activity at concentrations of 10 µM and 50 µM (Figure 2). 

The percentage of inhibition of SA sortase A activity was calculated and presented in Table 2. Maximal effect of flavuside B was observed during first 10 min of reaction and then it decreased slightly but remained statistically significant. 

### 2.3. Influence of Flavuside B on HaCaT Cells Co-Cultivated with SA

#### 2.3.1. The Viability of SA-Treated HaCaT Cells 

To understand whether flavuside B has a protective effect on the viability of SA-treated HaCaT cells, the cell permeability was detected by an LDH release assay and the cell metabolic activity was measured by an MTT assay. 

**Figure 3:**
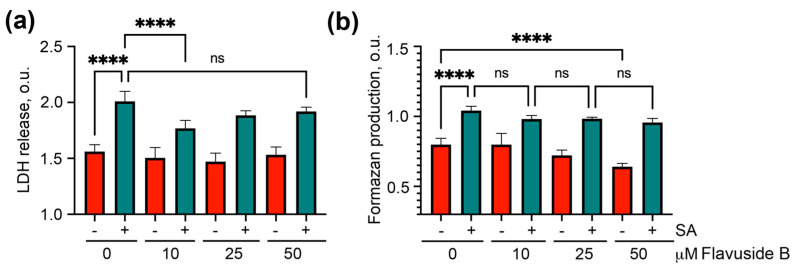
Influence of flavuside B at different concentrations on LDH release (**a**) and formazan production (**b**) in HaCaT cells co-cultivated with SA for 18 h. All experiments were performed in three independent replicates. The data presented as mean ± SEM. **** indicates the significant differences with *p* ≤ 0.0001, “ns” indicates that differences are not significant.

Co-cultivation of HaCaT cells with SA for 18 h enhanced the LDH release from HaCaT cells by 28.7% (Figure 3a). Flavuside B decreased the LDH release from SA-treated cells by 11.9%, 6.3% and 4.9% at concentrations of 10 µM, 25 µM, and 50 µM, respectively. Thus, treatment with flavuside B (10 µM) significantly diminished the additional LDH release from SA-treated cells by almost half (Figure 3a).

Co-cultivation of HaCaT cells with SA for 18 h overestimated the formazan reduction in the MTT assay by 31.6% (Figure 3b). Flavuside B at a concentration of 10 µM reduced the hyperproduction of formazan in SA-treated cells by 5.8% and the effects of flavuside B at concentrations of 25 µM and 50 µM were similar. However, a decrease of formazan production was observed in HaCaT cells treated with flavuside B at concentrations of 25 µM and 50 µM (Figure 3b). For this reason, flavuside B was used in the following experiments only at a concentration of 10 µM.

#### 2.3.2. Apoptotic Profile, Total Caspase Activity and Cell Cycle in SA-Treated HaCaT Cells 

The effect of flavuside B on the induction of apoptosis in SA-treated HaCaT cells was studied by flow cytometry using fluorescent annexin V conjugate as a fluorescent dye [14]. 

**Figure 4:**
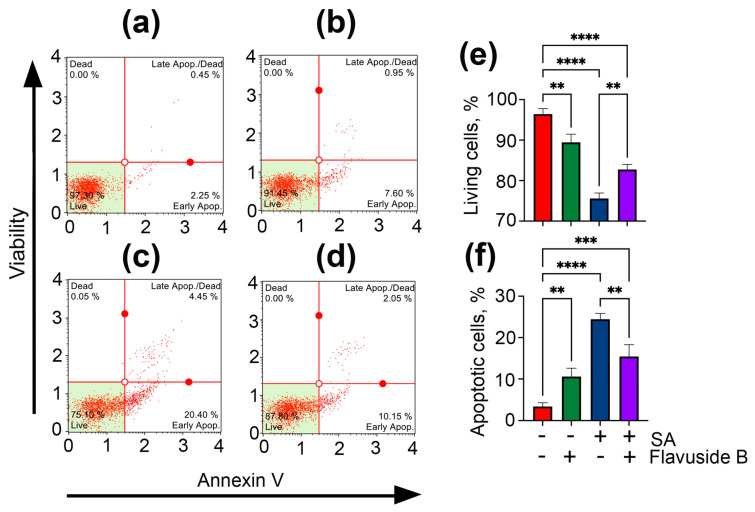
Influence of flavuside B on apoptosis profile in HaCaT cell co-cultivated with SA for 40 h. Data of flow cytometry of non-treated HaCaT cells (**a**), HaCaT cells treated with flavuside B (**b**), HaCaT cells treated with SA (**c**), HaCaT cells treated with SA and flavuside B (**d**) and summarized graphs (**e**,**f**). All experiments were performed in three independent replicates. The data presented as mean ± SEM. **** indicates the significant differences with *p* ≤ 0.0001; *** indicates the significant differences with *p* ≤ 0.005; ** indicates the significant differences with *p* ≤ 0.001.

Significant changes in the apoptotic profile of SA-treated HaCaT cells were observed after 40 h of co-culture (Figure 4d,e). The percentage of early apoptotic and late apoptotic cells in SA-treated HaCaT cells was 19.4% and 4.9%, respectively, in contrast to untreated cells with 3.1% and 0.4% of early apoptotic and late apoptotic cells, respectively. Flavuside B shown low influence on the apoptotic profile of HaCaT cells (Figure 4b,f) and decreased percentage of apoptotic cells in SA-treated HaCaT cells by more than half (Figure 4d,f). 

**Figure 5:**
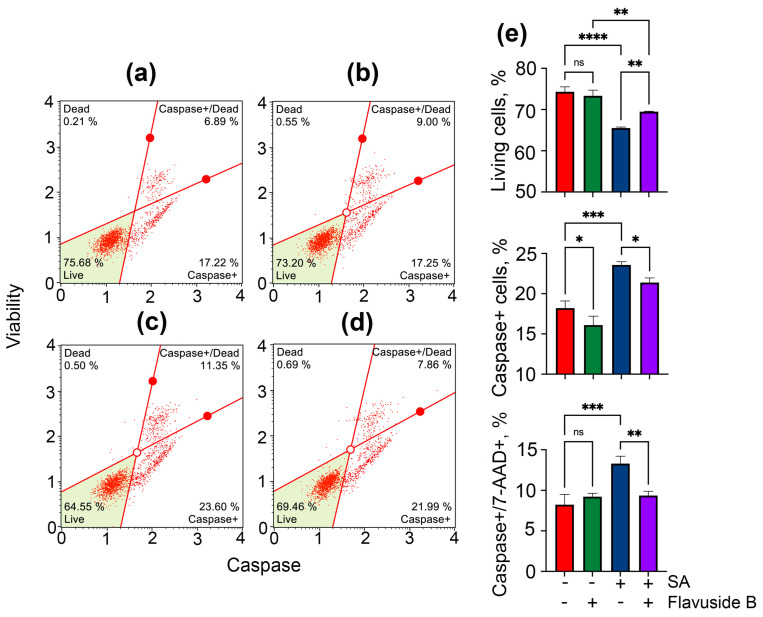
Influence of flavuside B on caspase activity in HaCaT cell co-cultivated with SA for 40 h. Data of flow cytometry of non-treated HaCaT cells (**a**), HaCaT cells treated with flavuside B (**b**), HaCaT cells treated with SA (**c**), HaCaT cells treated with SA and flavuside B (**d**) and summarized graph (**e**). All experiments were performed in three independent replicates. The data presented as mean ± SEM. **** indicates the significant differences with *p* ≤ 0.0001; *** indicates the significant differences with *p* ≤ 0.005; ** indicates the significant differences with *p* ≤ 0.001; * indicates the significant differences with *p* ≤ 0.05; “ns” indicates that differences are not significant.

The data on apoptosis were confirmed by experiments to determine the number of cells with activated caspases. The influence of flavuside B on the percentage of live (caspase−/7-AAD−), live with caspase activity (caspase+/7-AAD−), dead with caspase activity (caspase+/7-AAD−) and dead (caspase−/7-AAD+) in SA-treated HaCaT cells measured by Muse^®^ MultiCaspase Kit are presented in Figure 5. The kit utilizes a VAD-peptide derivatized with a fluorescent group and called Fluorescent-Labeled Inhibitor of Caspases (FLICA) [15]. The peptide binds to activated caspases with resulting fluorescent signal proportional to the number of active caspases in the cell. Fluorescent dye 7-AAD is used as a dead cell marker in this assay.

SA significantly increased the percentage of living cells exhibiting caspase activity and 7-AAD labeled cells exhibiting caspase activity by 26.0% and 44.6%, respectively (Figure 5c,e). Flavuside B did not cause caspase activation in HaCaT cells (Figure 5b,e). When SA-infected HaCaT cells were incubated with flavuside B, the percentage of live cells and 7-AAD labeled cells exhibiting caspase activity reduced by 10.4% and 32.0%, respectively (Figure 5d,e).

**Figure 6:**
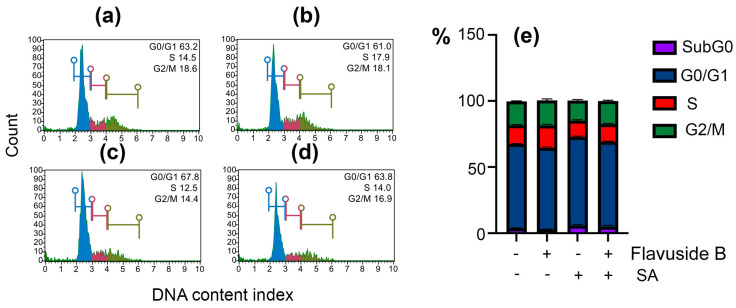
Influence of flavuside B on cell cycle in HaCaT cell co-cultivated with SA for 40 h. Data of flow cytometry of non-treated HaCaT cells (**a**), HaCaT cells treated with flavuside B (**b**), HaCaT cells treated with SA (**c**), HaCaT cells treated with SA and flavuside B (**d**) and summarized graph (**e**). All experiments were performed in three independent replicates. The data presented as mean ± SEM.

The effect of SA on the HaCaT cell cycle was minimal (Figure 6a,c,e). Nevertheless, a significant increase in G0/G1 cell amount and a reduce of S and G2/M cell amount as a result of SA infection were observed. Flavuside B alone did not induce significant changes in the HaCaT cell cycle (Figure 6b,e) and significantly diminished cell cycle alterations induced by SA (Figure 6d,e).

### 2.4. Influence on LPS-Induced HaCaT Cells

The protective influence of flavuside B on SA-treated HaCaT cells can be provided not only by direct suppression of SA growth and biofilm formation in SA-HaCaT co-culture, but also indirectly due to its anti-inflammatory effect. To induce an inflammatory in HaCaT cells they were treated with lipopolysaccharide (LPS) from *Escherichia coli*. LPS [16], similar to the cell wall components peptidoglycan and lipoteichoic acid from SA [17], increases intracellular nitric oxide (NO) level, as well as other inflammatory factors. So, the viability (LDH release) and NO level were detected in LPS-treated cells after incubation with flavuside B. Diaminofluorescein-FM diacetate (DAF-FM DA) as a cell-permeable fluorescent probe for high specific and sensitive detection of nitric oxide (NO) was used [18]. 

**Figure 7:**
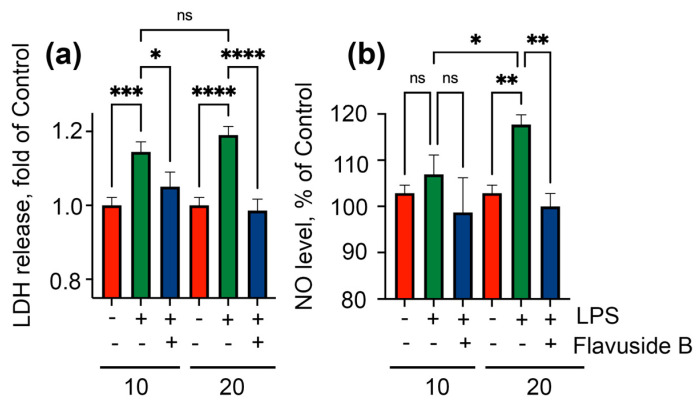
Influence of flavuside B on LDH release (**a**) and NO level (**b**) in LPS-treated HaCaT cells. All experiments were performed in three independent replicates. The data presented as mean ± SEM. **** indicates the significant differences with *p* ≤ 0.0001; *** indicates the significant differences with *p* ≤ 0.005; ** indicates the significant differences with *p* ≤ 0.001; * indicates the significant differences with *p* ≤ 0.05; “ns” indicates that differences are not significant.

Treatment of HaCaT cells with LPS at concentrations of 10 µg/mL and 20 µg/mL led to an enhance in the LDH release from cells by 14.4% and 19.0%, respectively (Figure 7a). Moreover, a significantly increase of NO level was found in HaCaT cells treated with 20 µg/mL of LPS (Figure 7b).

Flavuside B significantly reduced LDH release from both LPS-induced HaCaT cells to a control value and to baseline NO level in HaCaT cells administrated with high LPS concentration (Figure 7a,b). 

## 3. Discussion

The antimicrobial activity of cerebrosides (glycosphingolipids) isolated from various sources has been found long ago. It was reported about pinelloside from Chinese medicinal plant *Pinellia ternate* [19], two cerebrosides from *Euphorbia peplis* [20], fusaruside from endophytic fungus *Fusarium* sp. [21]. There is an opinion that the antimicrobial effect of cerebrosides is due to their competitive binding to pathogens instead of cell surface glycosphingolipids, with which pathogens interact with host cells [22]. Indeed, prevention of biofilm formation of methicillin-resistant strain of SA by cell adhesion inhibition was found for monohexosylceramides from *Rhizopus* species [23]. 

Earlier it was published that marine fungal cerebroside flavuside B visually inhibited the SA growth at a concentration (MIC) of 15.6 µg/mL [11], that is 20.7 µM. Our data are generally consistent with this report and provide a more accurate understanding that flavuside B inhibits SA growth by 28% at concentrations of 10.0 µM and 25.0 µM, and 50% inhibition occurs at 99.2 µM. It should be noted that Yang and co-authors used a SA suspension with a concentration of 1 × 10^5^ cells/mL while we used a SA suspension with a concentration 1 × 10^7^ CFU/mL as it is well-known and widespread.

We found that flavuside B inhibits SA biofilm formation, as well as the activity of sortase A enzyme. It is well known that microbial populations exist mainly as biofilms and biofilm formation is key for the functioning and pathogenic properties of SA colonies [24]. Moreover, the role of sortase A in the SA biofilm formation has been known for a long time [25]. It was recently reported that sortase A inhibition by two natural compounds can prevent the SA biofilm formation up to complete suppression [26]. Thus, the influence of flavuside B on biofilm formation may be directly related to the inhibition of sortase A activity by this cerebroside. However, since the sortase A inhibitory activity of flavuside B is not high enough (around 17% at a concentration of 10 μM), and this does not explain the effect of the investigated compound on the SA bacterial growth, other mechanisms may also contribute to the antimicrobial effects of flavuside B.

It was reported the inhibition of sortase A activity for various natural compounds, such as cinnamic acid and coumarin derivatives, flavonoids, 1,4-naphtoquinones, alkaloids, and several others [5]. Sortase A and SA bacterial growth inhibitory activities were found for β-sitosterol-3-O-glucopyranoside but were not found for its aglycon sitosterol which indicates the importance of the glucoside moiety [27]. However, the literature data about cerebrosides inhibited sortase A activity are unknown [28]. If so, the present work is the first report on the ability of cerebrosides to inhibit the activity of sortase A.

SA is often a component of the skin microbiome without causing harm or penetration deep into the body. An important role in this commensalism is played by glycosphingolipids of the skin barrier layer, which suppress the growth of pathogens. However, as soon as the protective properties of the skin are weakened, SA toxins have primarily a lytic effect on keratinocytes, destroying them in order to overcome the barrier and penetrate further [29]. As a result, SA caused an increase in the LDH release from infected cells, which was confirmed by our results. However, the cell viability measured by the MTT assay may contradict this. It was found that the MTT assay data for HaCaT cells treated with soluble factors from methicillin-resistant SA biofilm for 6 h was more than the MTT assay data for untreated cells [30]. It may consist of in the activation of the involved NADPH oxidase as a result of SA infection, which is a well-known mode for the reaction of keratinocytes on the bacterial infections [31]. On the other hand, it cannot be ruled out that the MTT overestimation resulted from the accelerated proliferation of HaCaT cells. The possibility of such a reaction on SA infection has been shown previously [32].

Programmed cell death is induced by SA in addition to the cell membrane perforation [2]. Apoptosis accompanied by the caspase activation, as well as the arrest cell cycle in G0/G1 phase were observed in our study during the co-cultivation of keratinocytes with SA. 

It was found that flavuside B protects keratinocytes from the toxic influence of SA. A decrease in the LDH release from SA-treated keratinocytes, as well as a diminishing of the number of cells with early and late apoptosis, a downregulation of caspase activity and a normalization of the cell cycle were observed because of the flavuside B administration. Obviously, it may be result of the direct antibacterial activity of flavuside B against SA. Moreover, the anti-inflammatory properties of flavuside B and its cytoprotective activity reported by us earlier [33] may play a significant role in the protection of HaCaT cells from SA toxicity. 

Chemically, cerebrosides are composed of hexose and ceramide moiety, which usually consist of a long-chain amino alcohol trivially called a “sphingoid base” (=sphingosine or sphingol) and an amide-linked long-chain fatty acid. Antimicrobial activity against Gram-positive and -negative bacteria has been reported for various sphingoid bases and fatty acids [7]. Moreover, a combined antimicrobial and anti-inflammatory activity has been reported for one of them, phytosphingosine [34]. The structure of flavuside B is similar to structures of fusaruside isolated from a endophytic fungus *Fusarium* sp. [21] and alternaroside B isolated from a halotolerant marine sediment-derived fungus *Alternaria raphani* [35], which possess antibacterial properties. In addition, fusaruside was found as anti-inflammatory agent [36]. All these fungal cerebrosides contain three double bonds in a ‘sphingoid base’ and same fatty acid residues. Probably, these structural features may be important for the flavuside B antibacterial and anti-inflammatory effects in our research. 

Thus, marine fungal cerebroside flavuside B protects HaCaT cells in in vitro model of SA skin infection. Flavuside B impaired the SA bacterial growth and the biofilm formation via sortase A activity inhibition and substantially eliminated such negative consequences caused by SA as cell permeabilization, induction caspase-depended apoptosis and the arrest cell cycle in G0/G1 phase. Apparently, this is caused, not only by the direct antibacterial effect, but also by the anti-inflammatory properties of flavuside B. The dual action of flavuside B may be highly effective in the treatment of bacterial skin lesions and will be studied in in vivo experiments in the future. 

## 4. Materials and Methods

### 4.1. Flavuside B

The isolation of flavuside B from sediment-derived fungus *Penicillium islandicum* (depth 50 m, Aniva Bay, the Sea of Okhotsk) and its structural investigation has been reported previously [12]. Before the bioassays, the compound was purified using published HPLC procedures and its chemical purity was confirmed by high resolution electrospray ionization mass spectrometry (HRESIMS). The compound was dissolved in DMSO (100%) at a concentration of 10 mM. This solution was used to obtain the required concentration of compound in the cell suspension so that the concentration of DMSO in the cell suspension did not exceed 1%.

### 4.2. Antimicrobial Activity against Staphilococcus aureus

#### 4.2.1. The Effect on Bacterial Growth

The antibacterial activity of flavuside B was evaluated as described previously [37] according to the method adopted by the Clinical and Laboratory Standards Institute (CLSI) with a slight modification of the medium.

The bacterial culture of *Staphylococcus aureus* ATCC 21027 (Collection of Marine Microorganisms PIBOC FEBRAS) was cultured in a Petri dish at 37 C for 24 h on solid medium (pepton—5.0 g/L, K_2_HPO_4_—0.2 g/L, glucose—1.0 g/L, MgSO_4_—0.05 g/L, yeast extract—1 g/L, agar—16.0 g/L, and distilled water—1.0 L). The pH of the medium was adjusted to 7.2–7.4 with NaOH solution.

The assays were performed in 96-well microplates in appropriate Mueller Hinton broth. The 90 µL of bacterial suspension (10^7^ CFU/mL), was then added to each well of the microplates.

Flavuside B was added to the wells in a volume of 20 µL diluted in PBS (DMSO concentration < 1%) at concentrations from 1.5 µM to 100 µM using two-fold dilution. Then, SA bacterial suspension was incubated with flavuside B at 37 °C for 18 h and their optical density was measured as absorbance at 600 nm with a microplate reader (BioTek, Winooski, VT, USA). Gentamicin was used as a positive control in concentration 1 mg/mL; 1% DMSO solution in PBS as a negative.

The results were presented as half-maximal effective concentration (EC_50_) and inhibition of bacterial growth in %. EC_50_ was calculated as concentration caused 50% inhibition of bacterial growth.

#### 4.2.2. The Effect on Biofilm Formation

The inhibition of the reducing biofilm formation and growth was assessed using the crystal violet biofilm assay as described [24]. Tryptic Soy Broth (TSB) with 2% glucose was inoculated with 10^7^ CFU/mL of SA overnight cultures. A total of 90µL of this cell suspension was then dispensed into 96-well microtiter plates containing 10 µL of flavuside B at concentrations from 1.5 µM to 100 µM using two-fold dilution. DMSO and PBS were used as solvents such as DMSO concentration did not exceed 1%. After 24 h growth at 37 °C the plates were washed with PBS to remove unbound cells and fixed ethanol. Next, the wells were stained with 0.1% crystal violet solution for 15 min at room temperature. At the completion of the incubation plates were washed 3 times with PBS and dried. Then the crystal violet dye from the biofilm was solubilized with 200 µL of ethanol. A total of 100 µL of this solution was then moved to a new microtiter plate for absorbance measurement at 595 nm. The results were reported as percent inhibition normalized to the wild type control.

### 4.3. The Effect on Sortase A Enzymatic Activity 

The enzymatic activity of sortase A from *Staphylococcus aureus* was determined using SensoLyte 520 Sortase A Activity Assay Kit *Fluorimetric* (AnaSpec AS-72229, AnaSpec, San Jose, CA, USA) in accordance with manufacturer’s instructions. DMSO at concentration of 0.1% was used as a control. Fluorescence was measured with the plate reader PHERAStar FS (BMG Labtech, Offenburg, Germany) for 60 min with a time interval of 5 min. The data were processed by MARS Data Analysis v. 3.01R2 (BMG Labtech, Offenburg, Germany). The results were presented as relative fluorescent units (RFU) and percent of control data.

### 4.4. HaCaT Cell Culture

The human keratinocytes cell line HaCaT were kindly provided by Prof. N. Fusenig, Cancer Research Centre, Heidelberg, Germany. The cells were cultured in DMEM medium (Biolot, St. Petersburg, Russia) containing 10% fetal bovine serum (Biolot, St. Petersburg, Russia) and 1% penicillin/streptomycin (Invitrogen, Carlsbad, CA, USA) at 37 °C in a humidified atmosphere with 5% (*v/v*) CO2. Initially, cells were incubated in cultural flasks until sub-confluent (~80%). For testing, HaCaT cells were seeded at concentrations of 6 × 10^4^ cells/mL in 6- (3 mL) or 96-well (180 µl) plates and experiments were started after 48 h.

### 4.5. Co-Cultivation of HaCaT Cells with SA

Co-cultivation of HaCaT cells with SA was carried out as described [13] with modifications [38]. HaCaT cells were seeded in 6- or 96-well plates for 48 h. Than culture medium in each well was changed with SA suspension (10^2^ CFU/mL) in DMEM medium without penicillin/streptomycin. Fresh DMEM medium without SA suspension was added in other wells as need. Flavuside B was added in wells after 1 h. HaCaT cells and SA were cultured at 37 °C in a humidified atmosphere with 5% (*v*/*v*) CO_2_ for 18 h or 40 h.

### 4.6. The Treatment of HaCaT Cells with Lipopolysaccharide (LPS) 

Lipopolysaccharide from *Escherichia coli* 055:B5 (Sigma-Aldrich, St. Louis, MO, USA) was used in this assay. HaCaT cells were seeded in 96-well plates for 48 h. Than culture medium in each well was changed with fresh full DMEM medium and LPS (1 mg/mL in PBS, stock solution) was added at different concentrations. The cells were cultured at 37 °C in a humidified atmosphere with 5% (*v/v*) CO_2_ for 24 h.

### 4.7. Lactate Dehydrogenase (LDH) Release Assay

After incubation, the plate was centrifuged at 250× *g* for 10 min and 100 µL of supernatant from each well was transferred into the corresponding wells of an optically clear 96-well plate. An equal volume of the reaction mixture (100 µL) from LDH Cytotoxicity Assay Kit (Abcam, Cambridge, UK) was added to each well and incubated for up to 30 min at room temperature. The absorbance of all samples was measured at λ = 490 nm using a Multiskan FC microplate photometer (Thermo Scientific, Waltham, MA, USA) and expressed in optical units (o.u.).

### 4.8. Formazan Production (MTT) Assay

After incubation, the cell viability was determined by the MTT (3-(4,5-dimethylthiazol-2-yl)-2,5-diphenyltetrazolium bromide) method according to the manufacturer’s instructions (Sigma-Aldrich, St. Louis, MO, USA). The absorbance of formed formazan was measured at λ = 570 nm using a Multiskan FC microplate photometer (Thermo Scientific, Waltham, MA, USA) and expressed in optical units (o.u.).

### 4.9. Flow Cytometry

#### 4.9.1. Apoptosis

After incubation, culture media was collected, and cells were washed by cold PBS twice and incubated with trypsin-EDTA solution for 1 min. The cell suspension was washed by centrifugated at 250× *g* for 4 min with cold PBS twice and then used for apoptosis detection by Muse^®^ Annexin V & Dead Cell Kit in accordance with manufacturer’s instructions (Luminex, Austin, TX, USA). The fluorescence was measured with Muse^®^ Cell Analyzer (Luminex, Austin, TX, USA) and data were processed by Muse 1.5 Analysis software (Luminex, Austin, TX, USA). The proportion of apoptotic cells was expressed as a percentage. 

#### 4.9.2. Total Caspase Activity

After incubation, culture media was collected, and cells were washed by cold PBS twice and incubated with trypsin-EDTA solution for 1 min. The cell suspension was washed by centrifugated at 250× *g* for 4 min with cold PBS twice and then used for caspase activity detection by Muse^®^ MultiCaspase Kit (caspase 1, 3, 4, 5, 6, 7, 8, and 9) in accordance with manufacturer’s instructions (Luminex, Austin, TX, USA). The fluorescence was measured with Muse^®^ Cell Analyzer (Luminex, Austin, TX, USA) and data were processed by Muse 1.5 Analysis software (Luminex, Austin, TX, USA). The proportion of the cells exhibiting caspase activity was expressed as a percentage. 

#### 4.9.3. Cell Cycle

After incubation, cells were trypsinized, harvested, washed with PBS and fixed with ice-cold 70% ethanol in a dropwise manner prior to storage at −20°C overnight. The cells were then washed with PBS, incubated with 200 μg/mL RNAse (PanReac, AppliChem, Germany) and 20 μg/mL of propidium iodide (Sigma-Aldrich, St. Louis, MO, USA), for 30 min at 37 °C and the DNA content was analyzed with Muse^®^ Cell Analyzer (Luminex, Austin, TX, USA). The data were processed by Muse 1.5 Analysis software (Luminex, Austin, TX, USA). The proportion of cells in each phase of the cell cycle was expressed as a percentage. 

### 4.10. The NO Level Estimation

After incubation, culture media was changed with PBS containing diaminofluorescein-FM diacetate (DAF FM-DA) at concentration of 10 μM (Sigma-Aldrich, St. Louis, MO, USA) and the plates were incubated for 40 min at 37 °C, then replaced with fresh PBS, and then incubated for an additional 30 min to allow complete de-esterification of the intracellular diacetates. The intensity of DAF FM-DA fluorescence was measured at λ_ex_ = 485 and λ_em_ = 520 nm using the plate reader PHERAstar FS (BMG Labtech, Offenburg, Germany). The data were processed by MARS Data Analysis v. 3.01R2 (BMG Labtech, Offenburg, Germany). The results were presented as a percent of control data [39].

### 4.11. Statistical Data Evaluation

All results were given as a mean ± standard error of the mean (SEM). General statistical analysis was performed with the use one-way ANOVA test followed by Tukey’s comparison test that was employed with the aid of GraphPad Prism 9.0.2 (GraphPad Software, Inc., San Diego, CA, USA). Differences were considered statistically significant at *p* < 0.05.

## Figures and Tables

**Table 1:** The effect of flavuside B on bacterial growth and biofilm formation in SA culture.

Concentration, µM	Inhibition of Bacterial Growth, %	Inhibition of Biofilm Formation, %
10.0	27.05 ± 1.48	28.93 ± 2.17
25.0	28.44 ± 3.15	23.54 ± 1.47
50.0	38.02 ± 2.31	25.86 ± 1.09
100.0	49.13 ± 2.05	28.32 ± 3.05

All experiments were performed in three independent replicates. The data presented as a mean ± SEM.

**Table 2:** The effect of flavuside B on SA sortase A enzymatic activity.

Time, min	Inhibition of SA Sortase A Activity, %	
	Flavuside B Concentration, µM	
	10.0	50.0
5	17.15 ± 0.75 *	15.43 ± 1.55 *
10	17.35 ± 1.12 *	12.29 ± 1.20 *
15	15.84 ± 1.17 *	9.75 ± 0.95 *
20	13.82 ± 1.55 *	8.12 ± 0.82 *
25	12.78 ± 1.47 *	8.63 ± 0.85 *
30	11.80 ± 1.32 *	9.81 ± 0.98 *
35	11.15 ± 1.21 *	10.13 ± 1.02 *
40	11.30 ± 1.12 *	8.87 ± 0.87 *
45	10.73 ± 1.22 *	7.23 ± 0.72 *
50	10.34 ± 1.13 *	6.10 ± 0.55 *
55	9.77 ± 1.1 2*	5.61 ± 0.52 *
60	10.16 ± 1.06 *	4.29 ± 0.39 *

All experiments were performed in three independent replicates. The data presented as a mean ± SEM. Asterisk * indicates that inhibition effect was statistically significant with *p* ≤ 0.05.

## Data Availability

Not applicable.
